# Effect of Experimental Pain and Visual Feedback on the Accuracy and Precision of Knee Joint Position Sense

**DOI:** 10.1155/prm/9328803

**Published:** 2025-04-10

**Authors:** Shaojun Liao, Lars Arendt-Nielsen, Kelun Wang, Rogerio Pessoto Hirata

**Affiliations:** ^1^Department of Orthopedics, Ningbo Medical Center LiHuiLi Hospital, Ningbo University, Ningbo, China; ^2^Center for Sensory-Motor Interaction (SMI), Department of Health Science and Technology, Aalborg University, Aalborg, Denmark; ^3^Department of Medical Gastroenterology, Mech-Sense, Aalborg University Hospital, Aalborg, Denmark; ^4^ExerciseTech Research Group, Department of Health Science and Technology, Aalborg University, Niels Jernes Vej 12, Aalborg, Denmark

**Keywords:** accuracy, experimental pain, knee reposition error, precision, visual feedback

## Abstract

**Objective:** To investigate the effects of experimental pain and visual feedback on the accuracy and precision of knee joint position sense following a period of motor training.

**Methods:** Forty healthy young subjects (age: 24.5 ± 3.6 years old) underwent an 8 day motor training. After the training, they were instructed to perform a knee reposition task before and after receiving an injection of either hypertonic (pain group) or isotonic (control group) saline into the infrapatellar fat pad of the left knee. The Visual Analog Scale (VAS) was recorded for both groups. In each condition, participants were instructed to extend their knee to three predetermined target positions (30°, 45°, and 60°) for 10 repetitions, both with visual feedback (VF) and without visual feedback (NVF). The accuracy and precision of the knee reposition task were measured before and after the injection. Accuracy was determined by calculating the mean difference between the target angle and the actual angle achieved, while precision was determined by calculating the standard deviation of all actual angles. Data were analyzed using two-way ANOVAs and independent-samples *t*-tests to compare the pain and control groups.

**Results:** The VAS were 4.14 ± 2.48 for the pain group and 0.83 ± 0.89 for the control group. There was a significant decrease in knee accuracy after the injection of hypertonic saline compared to movements before the injection during VF (*p*=0.009). The pain group showed significantly worse knee accuracy compared to the control group in the relative change of performance during VF (*p*=0.015).

**Conclusions:** This study demonstrates that experimental knee pain impairs the accuracy of joint position sense, even after a period of motor training. This could serve as a helpful cue for individuals with knee pain to pursue timely treatment, thereby reducing the risk of additional injury.

**Trial Registration:** ClinicalTrials.gov identifier: NCT04146311

## 1. Introduction

Knee pain is a common complaint that affects individuals of all ages and usually caused by injuries (such as a ruptured ligament or torn cartilage) or medical conditions (including arthritis, gout and infections) [1, 2]. Specifically, knee pain can originate from various anatomical structures, including the joint capsule, anterior synovium, medial and lateral retinaculum, patellar subchondral bone, patellar tendon, and infrapatellar fat pad [3]. The infrapatellar fat pad is a type of adipose tissue in the knee joint that is associated with an inflammatory reaction linked to knee osteoarthritis [4]. It was first described by Hoffa [5] in 1904 and is now commonly used to induce experimental knee pain through the injection of hypertonic saline [6–8]. Experimental knee pain has been shown to reduce the strength of knee muscles [9], affect the coordination of quadriceps muscles [10], and impair joint torque and muscle activation [11]. However, it is not fully understood how experimental knee pain can affect the accuracy and precision of joint position sense following a period of motor training.

Motor training has been suggested as an effective method for improving motor performance [12, 13]. However, it remains unclear how pain affects knee joint position sense after training under different sensory conditions, and whether there are differences in movement performance alterations between accuracy and precision. In joint reposition tasks, accuracy refers to the difference between the desired angle and the actual angle of motor performance, while precision refers to the variability among all actual angles [14]. A good performance in accuracy does not necessarily indicate a good performance in precision as well [15]. Therefore, it may be beneficial to assess both accuracy and precision in research [16].

Visual feedback (VF) is commonly used in motor task training as it provides information about task execution, which can enhance cognitive engagement and facilitate the consolidation of skill acquisition [17, 18]. Individuals may develop different motor strategies when executing tasks under different sensory feedback conditions [19]. When VF is provided, individuals can acquire knowledge of results and make immediate compensatory movements [20]. However, when relying solely on proprioceptive feedback, individuals can only predict the error between desired and actual movements through an internal brain model, which integrates afferent signals from the sensory system [21]. The influence of pain and VF on knee joint position sense were not fully understood.

Therefore, the objective of this study was to investigate the effect of pain and VF on the accuracy and precision of knee joint position sense after a period of motor training. We hypothesized that the accuracy and precision of knee position sense would decrease due to experimental pain even after motor training. Knee repositioning performance was better with VF compared with NVF.

## 2. Materials and Methods

### 2.1. Subjects

Forty young healthy subjects (26 men and 14 women; 24.5 ± 3.6 years) were randomly assigned to two even groups: the pain group (hypertonic saline) and the control group (isotonic saline). The sample size was calculated by GPower 3.1 with a risk of Type I and Type II errors of 5% and 20%, respectively, using a conservative estimate of 25% for intraindividual variation. Considering a drop-out rate of 20%, 20 subjects were needed in each group. The randomization was generated using a computer program. The code was opened after the statistical analysis. The study employed a double-blind design, where neither the participants nor the executor was aware of the type of solution received by participants. Inclusion criteria was healthy individuals with a dominant right leg. The leg dominance was self-reported by all subjects before they can be enrolled. Exclusion criteria included a history of knee injury or pathology, ongoing knee pain, previous mental or neurological illnesses, regular physical exercise (WHO guidelines of physical activity), current use of analgesics, pregnancy, lack of ability to cooperate, drug and alcohol addiction, or participation in other pain-related studies during the study period. The study was approved by the local Ethics Committee (N-20170080). All participants signed the informed consent before participating.

### 2.2. Experimental Procedure

**Figure 1:**
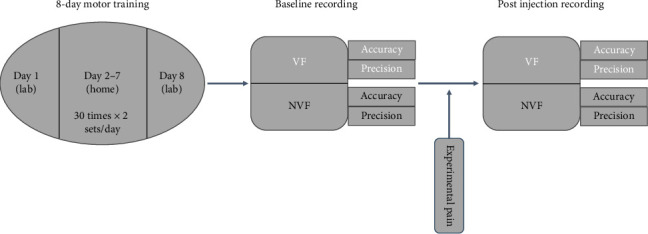
The flowchart of the experimental procedure of this study. Accuracy is represented by the absolute error to the target angle, while precision is represented by the standard deviation of actual angles. VF: with visual feedback, NVF: without visual feedback.

All participants underwent two sessions: Session 1, a baseline recording after 8 days of motor training, and Session 2, a recording after the injection of hypertonic or isotonic saline. The flowchart of the entire experimental procedure is illustrated in Figure 1.

The 8 day training involved a knee extension task to reach a target position of 45°. On the first day, participants received training in the lab using a 3D camera system, which allowed them to observe the real-time movements of their left knee on a screen. They then completed 6 days of training at home using a custom-made frame equipped with a blocker to assist their knee in stopping at the target angle of 45°. On the following day, they returned to the lab for additional training with the 3D camera system. The motor training consisted of two sets of knee extensions per day. Each set included 30 consecutive knee-reaching movements to the target position of 45° during VF. The speed of the movements was kept constant using a metronome set at 60 beats per minute, ensuring a consistent speed for each participant. Once all the training sessions were completed, participants underwent an assessment of the accuracy and precision of knee repositioning tasks under two visual conditions (VF and NVF). These measurements served as the baseline. Participants were seated on a table with their feet off the floor. Following the instructions of the 3D camera system, participants performed three target repositioning tasks (targets of 30°, 45°, and 60°). First, the investigator assisted the participants in reaching the target angle and maintained it for approximately 5 s. Then, the participants returned their leg to the initial position. Next, the participants were instructed to reposition their left knee to the three target angles for 10 trials each, both during VF and NVF. Both the target order (30°, 45°, and 60°) and feedback type (VF and NVF) were randomized. The speed of movement was regulated using the same metronome settings as in the training session. Finally, hypertonic or isotonic saline was injected into the infrapatellar fat pad of the left leg according to the pain and control groups. Then, the accuracy and precision of knee repositioning tasks were assessed again under two visual conditions (VF and NVF).

### 2.3. Knee Joint Angle

**Figure 2:**
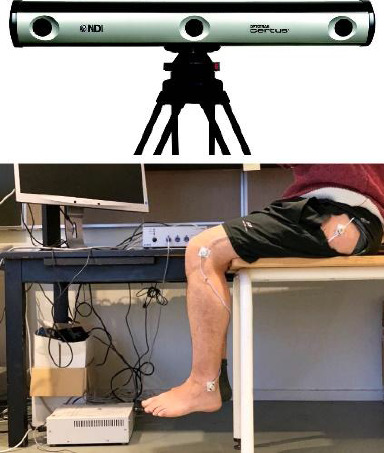
The 3D camera and three markers used in the present study. Three markers attached to the left leg following three anatomical points: The apex of the greater trochanter, the lateral femoral epicondyle, and the lateral malleolus.

The Optotrak Certus motion capture system (Northern Digital Inc, Ontario, Canada) was used to measure knee repositioning movements (sampling frequency of 4600 Hz and a resolution of 0.01 mm). Three markers were attached to the participants' left leg using double-sided stickers, following three anatomical points: the apex of the greater trochanter, the lateral femoral epicondyle, and the lateral malleolus. The line connecting the apex of the greater trochanter and the lateral femoral epicondyle represented the thigh axis, while the line connecting the lateral femoral epicondyle and the lateral malleolus represented the leg axis. The software NDI First Principles automatically and in real time calculated the angle formed by the two lines. The experimental setup is shown in Figure 2.

### 2.4. VF

The knee movements were performed with VF and NVF. During VF, participants were instructed to keep their eyes on the screen of the 3D camera system where they could see the real-time movement track of their leg. The screen displayed three markers in real time, as well as a small sign placed right at the target position to show participants exactly where the target position was. During NVF, participants were blindfolded while moving to the target position. All target angles (30°, 45°, and 60°) of the knee movements were recorded both during VF and NVF.

### 2.5. Experimental Knee Pain

The 40 participants were randomly divided into two groups: the pain group and the control group. The pain group was induced by injecting 0.25 mL of hypertonic saline (5%) into the infrapatellar fat pad of the left leg (medial to the patella tendon and proximal to the joint line). The control group received an equal amount of isotonic saline (0.9%) injected into the same site. The injection was administered by another tester who has been approved for the qualification. The injection site was determined by palpating the medial border of the patellar tendon, the medial condyle of the tibia, and the medial epicondyle of the femur. A needle (27 G, 19 mm, BD Microlance 3, Becton Dickinson, Ireland) was inserted at a 45° angle in a superolateral direction, with a depth of 15 mm. Before injection, the infusion site was sterilized with alcohol. Participants continuously scored the intensity of their pain on a 10 cm Visual Analog Scale (VAS), with the lower extreme labeled “no pain” and the upper extreme labeled “worst pain imaginable.” The VAS signal was sampled by a computer program every 2 s from the beginning of the infusion until the end of the experiment. There was no minimum VAS cutoff in the hypertonic saline group.

### 2.6. Statistical Analysis

The variability in amplitude of the movements was expressed in relation to the target position (accuracy) and as variations (precision). The accuracy of the motor behaviors was quantified by calculating the absolute error between the actual value (*x*) and the target value (*X*), expressed as *Accuracy = |X* − *x|.* The precision of the motor behaviors was determined by calculating the standard deviation (*σ*) relative to the mean value ($\bar{x}$). To assess the relative change of accuracy (RA) and precision (RP), the data were normalized to the values before injection, expressed as RA = A_after_ − A_before_ and RP = P_after_ − P_before_. The accuracy and precision data for the three angles were averaged for each participant before analysis. In addition, the data from before and after injection were transformed by log10 to ensure a normal distribution before being analyzed using a two-way ANOVA with the factors: sessions (2 levels: before injection and after injection) and visual conditions (2 levels: VF and NVF), with Bonferroni as the post hoc analysis. The independent-samples *t*-tests were used to compare the VAS score and the relative change of performance between the pain and control groups. The alpha value was set to 0.05.

## 3. Results

**Figure 3:**
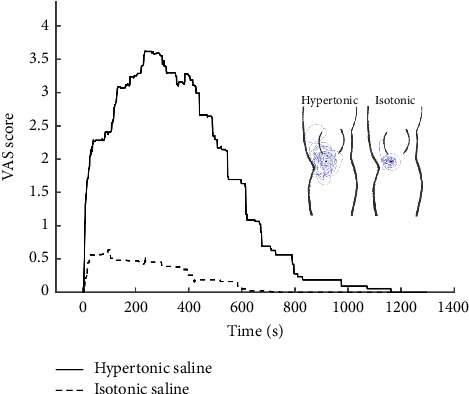
Visual Analog Scale (VAS) profile and pain area of 40 young healthy participants after the injection of hypertonic or isotonic saline into the infrapatellar fat pad. The VAS signal was sampled by a computer program every 2 s, and pain area drawings have been superimposed on the left leg.

Twenty subjects received an injection of hypertonic saline, while the other 20 subjects received isotonic saline. In the pain group, the mean pain intensity induced by the hypertonic saline was 4.14 ± 2.48, whereas in the control group, the mean pain intensity induced by the isotonic saline was 0.83 ± 0.89. The pain intensities after the injection of hypertonic and isotonic saline, as well as the drawing of the pain area, are shown in Figure 3. The means and standard deviations of average performance in the accuracy and precision before and after injection of hypertonic or isotonic saline are displayed in Table 1.

### 3.1. Effect of Injections

#### 3.1.1. Accuracy

**Figure 4:**
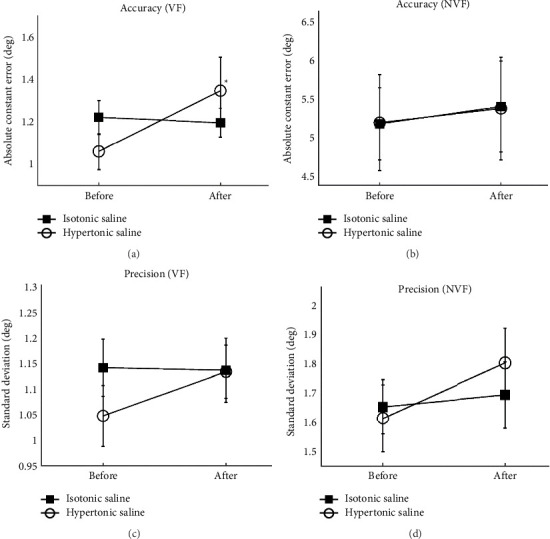
The accuracy (a-b) and precision (c-d) of knee joint position sense before and after injection of hypertonic or isotonic saline during with visual feedback (VF) and without visual feedback (NVF). ^∗^Indicates a significant difference after training compared to their baseline (*p* < 0.05).

There was no interaction between the visual conditions and injections. After the injection of hypertonic saline, the knee accuracy became significantly worse compared to the movement before injection during VF (2-way ANOVA, *F* = 0.048, *p*=0.009), whereas there was no significant difference in performance between before and after the injection during NVF (2-way ANOVA, *F* = 0.048, *p*=0.697) (Figures 4(a) and 4(b)). After the injection of isotonic saline, there were no significant differences in performance between before and after injection either during NF or NVF (2-way ANOVA, *F* = 0.451, *p* ≥ 0.545) (Figures 4(a) and 4(b)).

#### 3.1.2. Precision

There was no interaction between the visual conditions and injections. After the injection of either hypertonic or isotonic saline, there were no significant differences in performance after the injection compared to before injection either during NF or NVF (2-way ANOVA, hypertonic: *F* = 0.353, *p* ≥ 0.240 and isotonic: *F* = 0.178, *p* ≥ 0.663) (Figures 4(c) and 4(d)).

### 3.2. Relative Changes in Performance

#### 3.2.1. Accuracy

**Figure 5:**
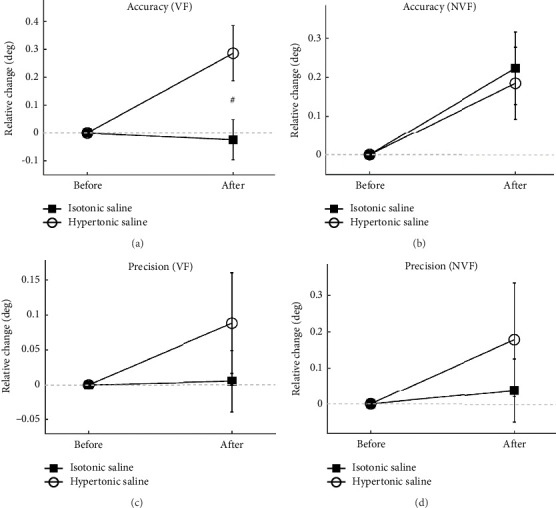
The relative change of accuracy (a-b) and precision (c-d) normalized to the baseline of knee repositioning performance before and after injection of hypertonic or isotonic saline during with visual feedback (VF) and without visual feedback (NVF). ^#^Indicates a significant difference after injection between the pain group and the control group (*p* < 0.05).

The pain group performed significantly worse than the control group in the relative change of movement during VF (*t*-test, *p* = 0.015). However, there was no significant difference between the pain and control groups during NVF (*t*-test, *p*=0.947) (Figures 5(a) and 5(b)).

#### 3.2.2. Precision

There was no significant difference in the relative change of precision between the pain and control groups either during NF or NVF (*t*-test, *p* ≥ 0.282) (Figures 5(c) and 5(d)).

## 4. Discussion

In this study, we compared the accuracy and precision of knee movements before and after injection as well as the differences between the pain and control groups. Significant differences were found when VF was provided.

### 4.1. Impact of the Experimental Pain

There was a significant decrease in accuracy, but not precision, after the injection of hypertonic saline compared to before the injection during the VF condition. In addition, there was a significant decrease in accuracy, but not precision, in the relative change of performance after the injection of hypertonic saline compared with isotonic saline during the same visual condition. This suggests a dissociation between accuracy and precision. Previous studies have demonstrated a negative impact of pain on joint position sense in the neck, lumbar, and extremities [22–24]. However, it remains unclear how experimental pain influences accuracy and precision disparately, and there is limited knowledge of the specific roles' accuracy and precision play in motor control. Accuracy refers to the extent of deviation from the true value, while precision relates to the reproducibility and repeatability of measurements [16]. In the present study, accuracy seemed to be more easily influenced by interventions, which may be due to a moderate level of pain perceived by participants [25]. Previous research has shown a positive correlation between pain intensity and cervical joint position error [26], suggesting that moderate pain may not be sufficient to reduce the precision of knee joint position sense. Further studies are needed to investigate potential differences between accuracy and precision in knee joint position sense at different levels of pain intensity. In addition, the standard deviation of VAS scores in the experimental group is large, indicating widely scattered data. This large variability in VAS scores could decrease the consistency in results of experimental pain, further affecting the accuracy and precision of knee joint position sense. In terms of accuracy, experimental pain in the present study reduced movement performance even after a period of motor training. It is well-known that motor training can improve movement performance [13, 27], while experimental pain can impair movement performance [28, 29]. In this study, 8 days' training may not be sufficient to form a learning effect. Further studies with higher intensity and longer training periods are needed to determine if they can mitigate the negative effects of pain.

Pain can impair motor behavior by affecting various parts of the nervous system, from spinal reflex circuits to the motor cortex [30]. Acute pain decrease the maximum contraction and endurance of muscles [31], impacting the motor units within the muscles [32] or the coordination between muscles [33], and affecting corticospinal excitability [34], resulting in poorer motor control [30]. By affecting force production, muscle activity patterns and kinematics [35], pain results in slower and less accurate movements [36]. It is a vicious cycle, leading to muscle inhibition or spasm near the painful site [37]. While the decline in accuracy may be linked to pain-induced limitations in the ability to perform the knee repositioning task, the reduction in maximum executive ability may also be a result of protective mechanisms triggered by experimental pain [38, 39]. In order to protect against further injury, the central nervous system adjusts its strategy by choosing the least painful and most appropriate movement pattern instead of optimizing performance [40]. This reduces stress on the painful tissue to prevent additional pain [41], but it also leads to a decrease in actual performance by increasing the “safety margin” for completing the task [38]. Moreover, individuals experiencing pain may perceive a greater effort required to reach a target, resulting in an overestimation of distance compared with those without pain [42]. In addition, the injection of hypertonic saline can impact the sensitivity of the muscle spindle through volume effects or local injury and have a direct effect on proprioceptive receptors around the injection site, leading to impairments in the process of proprioceptive signal transmission [43].

From the perspective of transfer of learning, the results of this study suggest that acquiring skills in a pain-free condition does not effectively transfer to a painful condition. Transfer typically occurs when there is a similar context or condition [44]. The more similar the tasks are, the higher the percentage of transfer [45]. Previous research demonstrated that induced pain during both the training and retention process showed no impact of pain on retention performance [46]. However, another study found a reduction in retention performance when participants were trained with pain while the retention process was pain free [47]. In the present study, pain was induced during the retention period while the training process was pain-free, and this negatively affected the accuracy of knee joint position sense. These results may suggest that motor skills acquired during a pain-free condition are consolidated in memory but do not transfer to a painful context.

### 4.2. Impact of Visual Conditions

It is interesting to note that all significant differences in accuracy occurred when VF was present. However, there is no research that clearly describes the relationship between VF and experimental pain. One possible explanation is that the baseline movements before injection, during VF, were much better than those during NVF. This suggests that there is a greater potential for a reduction in performance during VF after injection. The relatively worse performance during NVF may dilute the negative effect of experimental pain on the movement results. In addition, it has been shown that pain can cause an attentional shift of vision [20]. This may result in eye movements toward the painful area rather than toward VF [48, 49], leading to a bias in attention toward knee joint position sense and a decrease in accuracy. Further studies are needed to directly demonstrate how pain affects VF.

### 4.3. Methodological Limitations

In the current study, there is a lack of direct evidence to show variations in neuroplasticity in the central nervous system after training and injection. The dominance of the knee was determined solely by asking instead of using established tools, which could potentially compromise the robustness of the methodology. The outcomes of this study are limited to the particular training method employed in the experimental setup. Consequently, these results do not support any definitive conclusions regarding the overall effectiveness or ineffectiveness of rehabilitation and physical therapies for knee injuries. In addition, the relatively small sample size may be insufficient to uncover all possible evidence.

## 5. Conclusions

The present study reveals a dissociation between accuracy and precision. We demonstrate that experimental knee pain interferes with the accuracy of knee joint position sense when VF is provided. This may act as a useful prompt for individuals experiencing knee discomfort to seek prompt treatment, thus minimizing the chance of further injury. Future studies should strive to provide direct evidence of neuroplasticity in the motor cortex and changes in muscle activity in the knee caused by pain.

## Figures and Tables

**Table 1:** Mean values and standard deviations of accuracy and precision for the pain and control group in average knee repositioning performance of three target angles (30°, 45° and 60°) before and after injection, both with and without visual feedback.

Groups	Sessions	Visual feedback	Accuracy (°)	Precision (°)
Pain	Before injection	VF	1.057 ± 0.376^∗^	1.049 ± 0.275
		NVF	5.188 ± 2.768	1.603 ± 0.484
	After injection	VF	1.343 ± 0.704^∗^	1.137 ± 0.239
		NVF	5.372 ± 2.971	1.781 ± 0.495

Control	Before injection	VF	1.217 ± 0.358	1.145 ± 0.257
		NVF	5.173 ± 2.085	1.640 ± 0.392
	After injection	VF	1.193 ± 0.303	1.140 ± 0.286
		NVF	5.396 ± 2.628	1.678 ± 0.475

*Note:* VF, with visual feedback; NVF, without visual feedback.

^∗^Refer to significant differences between sessions.

## Data Availability

The data supporting the findings of this study are available upon reasonable request from the corresponding author.
